# Discrimination of volatiles in herbal formula Baizhu Shaoyao San before and after processing using needle trap device with multivariate data analysis

**DOI:** 10.1098/rsos.171987

**Published:** 2018-06-20

**Authors:** Yangyang Xu, Hao Cai, Gang Cao, Yu Duan, Ke Pei, Jia Zhou, Li Xie, Jiayu Zhao, Jing Liu, Xiaoqi Wang, Lin Shen

**Affiliations:** 1 School of Pharmacy, Nanjing University of Chinese Medicine, Nanjing 210023, People's Republic of China; 2 Engineering Center of State Ministry of Education for Standardization of Chinese Medicine Processing, Nanjing University of Chinese Medicine, Nanjing 210023, People's Republic of China; 3 School of Pharmacy, Zhejiang Chinese Medical University, Hangzhou 310053, People's Republic of China; 4 Institute of Pharmaceutical and Food Engineering, Shanxi University of Traditional Chinese Medicine, Taiyuan 030024, People's Republic of China

**Keywords:** Baizhu Shaoyao San, traditional Chinese medicine processing, needle trap device, GC–MS, multivariate statistical analysis, chemical markers

## Abstract

To characterize the chemical differences of volatile components between crude and processed Baizhu Shaoyao San (BSS), a classical Chinese herbal formula that is widely applied in the treatment of gastrointestinal diseases, we developed a gas chromatography–mass spectrometry-based needle trap device combined with multivariate data analysis to globally profile volatile components and rapidly identify differentiating chemical markers. Using a triple-bed needle packed with Carbopack X, DVB and Carboxen 1000 sorbents, we identified 121 and 123 compounds, respectively, in crude and processed BSS. According to the results of principal component analysis and orthogonal partial least-squares discriminant analysis, crude and processed BSS were successfully distinguished into two groups with good fitting and predicting parameters. Furthermore, 21 compounds were identified and adopted as potential markers that could be employed to quickly differentiate these two types of samples using S-PLOT and variable importance in projection analyses. The established method can be applied to explain the chemical transformation of Chinese medicine processing in BSS and further control the quality and understand the processing mechanism of Chinese herbal formulae. Besides, the triple-bed needle selected and optimized in this study can provide a valuable reference for other plant researches with similar components. Furthermore, the systematic research on compound identification and marker discrimination of the complex components in crude and processed BSS could work as an example for other similar studies, such as composition changes in one plant during different growth periods, botanical characters of different medicinal parts in same kind of medicinal herbs and quality identification of one species of medicinal herb from different regions.

## 1. Introduction

Baizhu Shaoyao San (BSS), a classic herbal formula in traditional Chinese medicine (TCM), is generally used for treating gastrointestinal diseases. BSS is composed of Atractylodis Macrocephalae Rhizoma (Asteraceae), Paeoniae Radix Alba (Ranunculaceae), Citri Reticulatae Pericarpium (Rutaceae), and Saposhnikoviae Radix (Umbelliferae) at a ratio of 6 : 4 : 3 : 4. As first recorded in ‘Dan-Xi-Xin-Fa', a comprehensive medical treatise edited by distinguished physician Zhen-heng Zhu (AD 1281–1358) in the Yuan Dynasty, BSS was applied as a typical mediative formula to soften the liver and tonify the spleen, as well as eliminate dampness and relieve diarrhoea. Clinically, in China, BSS has been widely prescribed to treat acute and chronic intestinal inflammation, painful diarrhoea and diarrhoea-predominant irritable bowel syndrome [1,2]. The clinical efficacies of BSS are the synergistic results of some bioactive substances contained in BSS, such as volatile oils, sesquiterpene lactones, monoterpene glycosides, flavonoids and flavonoid glycosides, coumarins, chromones and other ingredients [3–6]. Previous studies have suggested that the volatile components of medicinal herbs contained in BSS exhibit gastrointestinal peristalsis promotion, anti-inflammation, suppression of tumour cell proliferation, anti-oxidation and anti-microbial effects [7–10]. According to the theory of TCM and based on Chinese Pharmacopoeia, three medicinal herbs contained in BSS must be processed in specific processing methods to enhance the curative efficacy of the whole formula and reduce side effects. These medicinal herbs are Atractylodis Macrocephalae Rhizoma (stir-frying with honey-processed wheat bran), Paeoniae Radix Alba (stir-frying with wheat bran) and Citri Reticulatae Pericarpium (stir-frying without any auxiliary materials). The chemical compositions of volatile oils significantly change during stir-frying, causing different treatment effects [11–13]. Previous processing studies have observed that significant transformations occur in the volatile components of Atractylodis Macrocephalae Rhizoma [14], Paeoniae Radix Alba [15] and Citri Reticulatae Pericarpium [16] during processing. However, no study has been performed on the comprehensive mining of global volatile characteristics between crude and processed BSS, causing inconsistencies in the clinical medication. In addition, non-standard prescriptions of mixing crude and processed products also exist in other places, thereby weakening the curative effects or causing toxic reactions. Therefore, a simple and reliable analytical method to evaluate the processing in BSS is necessary for quality control and guaranteed safety in clinical applications.

The purpose of the study is to develop a simple and convenient technique to quickly extract, analyse, and classify the volatile constituents in crude and processed BSS. An efficient approach to extract and determine the overall concentration of volatile compounds is the needle trap device (NTD), which is a powerful tool for preparing semi-volatile and volatile compounds [17,18]. NTD consists of a stainless-steel needle containing various sorbent fillers, a vacuum pumping enrichment system with a heating chamber and a gas-tight syringe. NTD integrates the functions of sample preparation, sampling and sample preconcentration in one step, and can conveniently be coupled with other analytical methods with high sensitivity, such as gas chromatography–mass spectrometry (GC–MS) and liquid chromatography–mass spectrometry [19,20]. In addition, NTD reduces the thermal decomposition of compounds and waste of time and resources compared with traditional steam distillation extraction, and increases the detection range and sensitivity of compounds from low to high boiling point compared with conventional static headspace extraction [21]. Moreover, a specific packing material or a combination of several packing materials coating the inside of the extraction needle endows the NTD with superb sampling selectivity and enables it to adsorb the target volatile compounds and reduce the matrix effects [22]. In recent years, NTD was successfully applied to analyse volatile organic components in the chemical industry, food science and environmental monitoring fields [23–25]; however, it has been seldom employed in the study of TCM processing.

Multivariate data analysis enables comprehensive profiling and classification of several samples containing numerous and complicated data; thus, it has been extensively used for the analytical research of TCM, e.g. monitoring the chemical changes in different cultivation areas and ages [26], classifying the quality grades of same species [27] and exploring the marker compounds of variant processing methods [28]. The chemometrics tools for statistical strategy, such as principal component analysis (PCA), partial least-squares-discriminant analysis and orthogonal partial least-squares-discriminant analysis (OPLS-DA), are gradually becoming increasingly important because they supply efficient and stable patterns for analysis, modelling, and interpretation of the extremely complex original dataset of biology and chemistry. Moreover, these kinds of chemometrics methods provide convenient, fast and reliable data processing modes based on data dimensionality reduction while retaining the information of variables as completely as possible.

In this study, NTD coupled with GC–MS was applied to extract and analyse the volatile compounds in crude and processed BSS for the first time. Multivariate data analysis consisting of PCA and OPLS-DA based on the spectra data of chemical information was performed to overview and discriminate the potential marker compounds between crude and processed BSS. This method of study will immensely facilitate the analysis of volatile components that can be used to control the quality and reveal the chemical transformation of processing among other TCM formulae.

## 2. Experimental

### 2.1. Samples and materials

Herbal materials of crude Atractylodis Macrocephalae Rhizoma, Paeoniae Radix Alba, Citri Reticulatae Pericarpium and Saposhnikoviae Radix were all purchased from Bozhou Jingwan TCM Pieces Factory in China and were authenticated by Professor Hao Cai. Processed Atractylodis Macrocephalae Rhizoma, processed Paeoniae Radix Alba and processed Citri Reticulatae Pericarpium were prepared according to the processing standards described in Chinese Pharmacopoeia [29]. The voucher specimens were deposited in School of Pharmacy, Nanjing University of Chinese Medicine (Nanjing, China).

The following materials were purchased from PAS Technology (Magdala, Germany): 22-gauge stainless-steel needle (51 mm × 0.40 mm i.d., 0.72 mm o.d.) with a straight cavity through both ends packing with 0.7 cm of Carbopack X (60/80 mesh), 0.7 cm of DVB (60/80 mesh), 0.7 cm of Carboxen 1000 (60/80 mesh) and an NT-Case (42.5 cm × 28.4 cm × 15.5 cm) multifunctional sampling device.

### 2.2. Sampling by needle trap device

**Figure 1.**
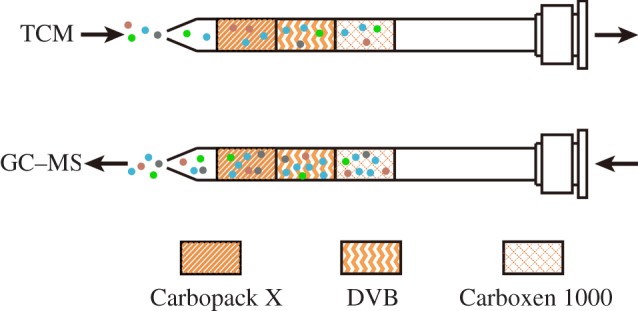
Illustration of adsorption and desorption process via triple-bed packed stainless needle in this experiment.

Prior to each sample extraction, the stainless-steel needle of NTD was conditioned in the GC injector at 250°C for 10 min with a constant helium flow to eliminate impure substances. For crude BSS, fine powders (sifted through an 80-mesh sieve) of crude Atractylodis Macrocephalae Rhizoma (30 mg), crude Paeoniae Radix Alba (20 mg), crude Citri Reticulatae Pericarpium (15 mg) and crude Saposhnikoviae Radix (20 mg) were accurately weighed, mixed and transferred into a 20 ml headspace vial. Afterward, 15 µl deionized water was added into the headspace vial to promote desorption and vaporization of volatile analytes from the matrix. The vial was tightly sealed with a polytetrafluoroethylene septum cap, placed in the heating chamber mounted on the NTD and heated at 80°C for 20 min. The needle was inserted into the vial septum and was exposed sufficiently to the headspace of the sample powders during the extraction. The needle was then connected to the vacuum pumping enrichment system of NTD to aspirate the headspace gas through the needle, by which the target compounds of the samples were adsorbed into the sorbent fillers. Pump flow rate and aspiration volume were set to 2 ml min^−1^ and 30 ml, respectively. After sampling, the needle was removed from the NTD, connected to a 1.0 ml gas-tight syringe, and inserted into the GC injector immediately to thermally desorb the trapped analytes at 250°C for 1 min. The schematic representation of the adsorption and desorption process via the triple-bed packed needle is illustrated in figure 1.

Similarly, the processed BSS sampling by the NTD was conducted using the same procedure as that of crude BSS using processed Atractylodis Macrocephalae Rhizoma, processed Paeoniae Radix Alba, processed Citri Reticulatae Pericarpium and crude Saposhnikoviae Radix.

### 2.3. Gas chromatography–mass spectrometry analysis

The analyses of volatile components of crude and processed BSS were performed with an Agilent 7890A-5975C GC-MS system (Agilent Technologies, Santa Clara, CA, USA). The separation was performed on a HP-VOC capillary column (60 m × 0.32 mm i.d.; 1.8 µm film thickness; Agilent Technologies). The injection port temperature was 250°C, GC–MS interface temperature was 250°C, ion source temperature was 230°C, MS quadrupole temperature was 150°C, electron ionization voltage was 70 eV, solvent delay was 5 min and splitless mode was used. Ultra high purity helium (99.999%) was used as the carrier gas at a permanent flow rate of 1 ml min^−1^. MS analysis was performed in full scan mode with a scan range of *m/z* 40 to 550. The oven temperature programme was initially held at 50°C for 1 min, then ramped to 200°C at a rate of 15°C min^−1^, and subsequently ramped to 250°C at a rate of 2°C min^−1^, and then ramped to 260°C at a rate of 10°C min^−1^, and finally held at 260°C for 5 min. Raw data were processed using MSD ChemStation E. 02. 02. 1431 (Agilent Technologies), where the peaks of all samples were aligned and integrated. Automatic mass spectral deconvolution and identification system (AMDIS) was employed to deconvolute GC–MS data file for better identifying the target compounds. Identification of the compounds was carried out by matching the experimental spectra with NIST14 MS spectral library and supported by GC Kovats retention index values.

### 2.4. Multivariate data analysis

The processed three-dimensional data matrices consisting of retention time, peak area and sample name were generated and imported into SIMCA-P 14.1 (Umetrics, Umea, Sweden) for multivariate data analysis. To eliminate the deviation caused by the large range of variable values, all the data underwent linear transformation and Pareto scaling (variables were weighted by 1/√s.d.) before unsupervised PCA and supervised OPLS-DA. To evaluate the quality of the established models, we calculated the goodness of fitting parameter (*R*^2^*X*), the ratio of the variance of the response variable explicated by the model (*R*^2^*Y*) and the ability of predicting new data (*Q*^2^) using a seven-round internal cross-validation of the original data using the default settings of SIMCA-P 14.1. In this multivariate data analysis strategy, PCA initially served to cluster all the samples and eliminate the outliers. Afterward, OPLS-DA was subsequently performed to produce visualization results to simplify the procedure for globally classifying and differentiating the targeted profiling obtained from the GC–MS spectra data. The S-plot of OPLS-DA was then employed to better visualize the contribution of chemical components toward the discrimination between crude and processed BSS. The S-plot was a scatter plot consisting of the covariance (*p*) versus correlation (*p*(corr)) vectors of the predictive components and helped identify variant markers between classes. This component, which had a high *p* value, indicated high model contribution, whereas a high *p*(corr) value indicated a high model reliability. A *p*(corr) greater than 0.5 combined with *p* > 0.1 was applied as a cutoff value to acquire the marker compounds in this paper. As a supplement and validation, the variable importance in the projection (VIP) value was applied to evaluate the variable contribution and determine the marker compounds. Variables with VIP values larger than 1 were considered important components that were responsible for model classification.

## 3. Results and discussion

### 3.1. Method development and validation

BSS, which consists of four herbal medicines of different families and genera, is an extremely complex matrix that contains numerous volatile components and has a low to relatively high boiling range. Therefore, simultaneous extraction and determination of these various types of volatile compounds in BSS using NTD coupled with GC–MS is challenging. To extract a wide range of volatile compounds in this investigation, we applied and tested different types of sorbents in the needle trap. The extraction efficacies of volatile components in BSS were compared using a double-bed needle packing Tenax and polydimethylsiloxane (PDMS), a triple-bed needle packing PDMS, DVB and Carboxen 1000, and a triple-bed needle packing Carbopack X, DVB and Carboxen 1000 under the same conditions. The results indicated that the triple-bed needle packing Carbopack X, DVB and Carboxen 1000 was highly suitable for the analysis of BSS due to its improved extraction ability for the low-, medium- and high-boiling point compounds, and low peak broadening compared with the other needles. The heating temperature of the NTD method was also a significant parameter that influenced the extraction effect [30]. Heating temperatures ranging from 60°C to 90°C were investigated in this experiment using peak numbers, areas and resolutions as optimization criteria. The amounts and intensities of the peaks were markedly increased when heating temperature increased from 60°C to 90°C. However, the peak intensities of the low-boiling-point compounds and the peak resolutions of all the peaks significantly decreased from 80°C to 90°C, perhaps due to thermal degradation and desorption when temperature was too high. As a result, 80°C was finally selected as the optimal heating temperature of NTD.

Robustness and ruggedness of this instrumental analysis method were evaluated using continuous six-time parallel analysis of one sample. Blank sample was used to investigate the background interference of this method, and little interference was observed according to the result. The profile of the blank sample is shown in electronic supplementary material, figure S1. Relative standard deviation (RSD) values of retention times and peak areas were evaluated to examine the repeatability of the sample NTD method. Resulting data showed that the RSD values of retention times and peak areas of corresponding main peaks in these six samples were less than 0.1% and 10%, which demonstrated that the developed NTD method was robust and had superb stability and repeatability.

### 3.2. Gas chromatography–mass spectrometry analysis of volatile components

**Figure 2.**
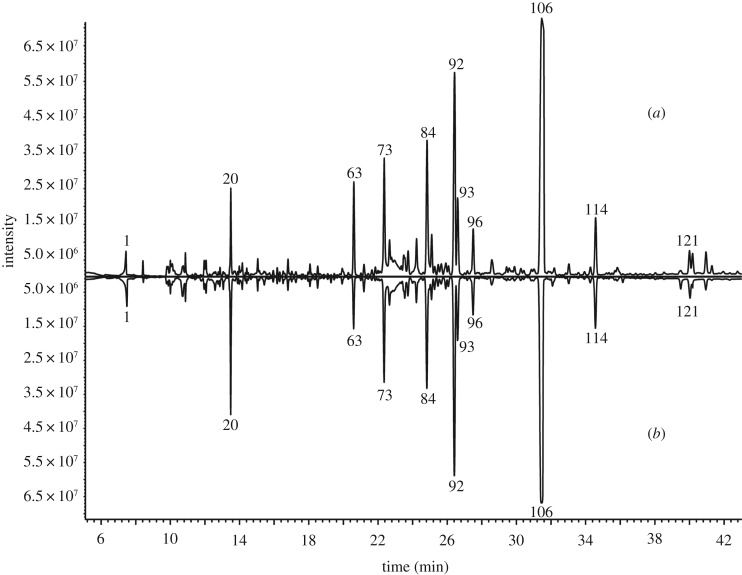
Typical profiles of volatiles in crude and processed BSS obtained by GC–MS: (*a*) crude BSS, (*b*) processed BSS.

Under the current NTD and GC–MS methods described earlier, 121 and 123 compounds, respectively representing 92.64% and 93.08% of the total volatile composition from crude and processed BSS, were separated and identified with match quality greater than 800 using AMDIS with the simple mode in Agilent MSD ChemStation. The identification of volatiles was confirmed by matching the experimental mass spectral data with NIST14 mass spectral library and was further elucidated using the Wiley 6 library. The chemical composition of volatiles from crude and processed BSS and their corresponding peak areas are summarized in table 1. Figure 2 shows the typical profiles of crude and processed BSS obtained through GC–MS, wherein the peaks with relatively high intensities are marked with serial numbers. On the basis of the structure of the volatiles, the components of crude BSS were divided into five groups: acids and esters (7.31%), aldehydes and ketones (8.15%), terpene hydrocarbons (41.77%), oxygenated terpenes (33.25%) and others (2.16%). Equally, the components of processed BSS were classified into five groups: acids and esters (7.58%), aldehydes and ketones (8.02%), terpene hydrocarbons (42.99%), oxygenated terpenes (31.56%) and others (2.93%).

**Table 1.** Identification of volatile compounds in crude and processed BSS by GC–MS (*n* = 6).

				measured area (×10^8)^	
no.	*T*_R_ (min)	compound name	formula	crude BSS	processed BSS
1	7.38	acetic acid	C_2_H_4_O_2_	3.33 ± 0.35	3.37 ± 0.30
2	9.86	(R,R)-2,3-butanediol	C_4_H_10_O_2_	0.78 ± 0.17	0.81 ± 0.13
3	9.99	(S,S)-2,3-butanediol	C_4_H_10_O_2_	1.82 ± 0.24	1.97 ± 0.22
4	10.13	isovaleric acid	C_5_H_10_O_2_	3.06 ± 0.84	2.86 ± 0.51
5	10.27	2-methylbutanoic acid	C_5_H_10_O_2_	0.81 ± 0.20	0.75 ± 0.22
6	10.70	furfural	C_5_H_4_O_2_	1.06 ± 0.28	5.85 ± 0.85
7	10.87	methyl *N*-hydroxylbenzenecarboximidoate	C_8_H_9_NO_2_	2.43 ± 0.28	2.86 ± 0.38
8	11.28	angelic acid	C_5_H_8_O_2_	0.38 ± 0.07	0.38 ± 0.09
9	11.44	heptanal	C_7_H_14_O	0.48 ± 0.04	0.50 ± 0.09
10	11.76	2,6-dimethylpyrazine	C_6_H_8_N_2_	0.33 ± 0.03	0.93 ± 0.03
11	12.07	hexanoic acid	C_6_H_12_O_2_	2.24 ± 0.18	2.66 ± 0.27
12	12.27	1-heptanol	C_7_H_16_O	0.30 ± 0.03	0.34 ± 0.04
13	12.52	phenol	C_6_H_6_O	0.73 ± 0.03	0.72 ± 0.08
14	12.59	5-methyl-2-furfural	C_6_H_6_O_2_	—	0.86 ± 0.15
15	12.62	6-methyl-5-hepten-2-ketone	C_8_H_14_O	0.85 ± 0.04	0.84 ± 0.03
16	12.78	benzaldehyde	C_7_H_6_O	0.49 ± 0.03	0.52 ± 0.06
17	12.87	octanal	C_8_H_16_O	0.97 ± 0.08	0.98 ± 0.12
18	13.13	cosmene	C_10_H_14_	0.50 ± 0.04	0.71 ± 0.09
19	13.29	4-carene	C_10_H_16_	0.21 ± 0.02	0.20 ± 0.03
20	13.50	D-limonene	C_10_H_16_	10.71 ± 1.18	15.89 ± 1.15
21	13.70	benzyl alcohol	C_7_H_8_O	0.75 ± 0.06	0.68 ± 0.11
22	13.75	1-octanol	C_8_H_18_O	0.56 ± 0.07	0.54 ± 0.06
23	13.89	γ-terpinene	C_10_H_16_	0.53 ± 0.06	0.64 ± 0.03
24	13.99	benzeneacetaldehyde	C_8_H_8_O	1.05 ± 0.07	1.06 ± 0.11
25	14.34	linalool	C_10_H_18_O	0.31 ± 0.01	0.33 ± 0.02
26	14.42	nonanal	C_9_H_18_O	0.95 ± 0.13	0.83 ± 0.11
27	14.50	*p*-cymenene	C_10_H_12_	0.38 ± 0.04	0.41 ± 0.05
28	14.66	guaicolina	C_7_H_8_O_2_	0.29 ± 0.05	0.33 ± 0.07
29	14.76	3-methylbenzaldehyde	C_8_H_8_O	0.33 ± 0.04	0.10 ± 0.06
30	14.92	1,3,8-*p*-menthatriene	C_10_H_14_	0.45 ± 0.15	0.18 ± 0.13
31	15.06	octanoic acid	C_8_H_16_O_2_	2.04 ± 0.25	1.90 ± 0.41
32	15.43	benzoic acid	C_7_H_6_O_2_	1.43 ± 0.22	1.89 ± 0.21
33	15.73	ethyl octanoate	C_10_H_20_O_2_	0.73 ± 0.04	0.68 ± 0.05
34	15.95	(+)-nopinone	C_9_H_14_O	0.78 ± 0.04	0.77 ± 0.06
35	16.18	decanal	C_10_H_20_O	1.04 ± 0.16	0.85 ± 0.11
36	16.32	4-terpinenol	C_10_H_18_O	0.49 ± 0.02	0.61 ± 0.06
37	16.52	α-terpineol	C_10_H_18_O	0.61 ± 0.06	0.90 ± 0.16
38	16.60	4-methylacetophenone	C_9_H_10_O	0.38 ± 0.07	0.38 ± 0.06
39	16.80	myrtanal	C_10_H_16_O	2.05 ± 0.23	1.88 ± 0.12
40	16.89	nonanoic acid	C_9_H_18_O_2_	0.38 ± 0.04	0.45 ± 0.07
41	17.02	(Z)-carveol	C_10_H_16_O	0.76 ± 0.10	0.76 ± 0.08
42	17.45	(E)-2-decenal	C_10_H_18_O	0.16 ± 0.04	0.14 ± 0.02
43	17.59	4-methyleneisophorone	C_10_H_14_O	0.23 ± 0.02	0.33 ± 0.04
44	17.67	ethyl nonylate	C_11_H_22_O_2_	0.29 ± 0.04	0.20 ± 0.07
45	17.80	carvone	C_10_H_14_O	0.15 ± 0.02	0.28 ± 0.04
46	17.96	thymol	C_10_H_14_O	0.50 ± 0.04	0.60 ± 0.08
47	18.07	(−)-myrtanol	C_10_H_18_O	1.38 ± 0.10	1.32 ± 0.17
48	18.17	4-isopropylanisole	C_10_H_14_O	0.30 ± 0.01	0.34 ± 0.03
49	18.28	undecanal	C_11_H_22_O	0.16 ± 0.01	0.17 ± 0.02
50	18.37	3-methyl-4-isopropylphenol	C_10_H_14_O	0.22 ± 0.04	0.26 ± 0.05
51	18.52	carvacrol	C_10_H_14_O	1.21 ± 0.06	1.60 ± 0.19
52	18.66	cuminic alcohol	C_10_H_14_O	0.52 ± 0.10	0.48 ± 0.09
53	18.87	perillic alcohol	C_10_H_16_O	0.40 ± 0.02	0.41 ± 0.05
54	19.09	decanoic acid	C_10_H_20_O_2_	0.45 ± 0.05	0.45 ± 0.14
55	19.29	2-methoxy-4-vinylphenol	C_9_H_10_O_2_	0.20 ± 0.02	0.88 ± 0.13
56	19.47	nerol acetate	C_12_H_20_O_2_	0.31 ± 0.02	0.30 ± 0.02
57	19.65	δ-eIemene	C_15_H_24_	0.26 ± 0.05	0.16 ± 0.03
58	19.80	silphiperfol-5-ene	C_15_H_24_	0.49 ± 0.14	0.15 ± 0.07
59	19.94	geranyl acetate	C_12_H_20_O_2_	0.92 ± 0.27	0.50 ± 0.13
60	19.99	α-cubebene	C_15_H_24_	1.12 ± 0.15	0.79 ± 0.09
61	20.29	eugenol	C_10_H_12_O_2_	0.63 ± 0.12	0.44 ± 0.07
62	20.37	decyl acetate	C_12_H_24_O_2_	0.23 ± 0.05	0.13 ± 0.02
63	20.61	7,7-dimethyl-1-vinylbicyclo[2.2.1]heptan-2-one	C_11_H_16_O	14.24 ± 1.90	7.92 ± 1.45
64	20.76	lauraldehyde	C_12_H_24_O	0.22 ± 0.03	0.13 ± 0.03
65	20.86	ylangene	C_15_H_24_	0.13 ± 0.01	0.09 ± 0.01
66	21.01	α-copaene	C_15_H_24_	0.49 ± 0.08	0.46 ± 0.09
67	21.19	β-elemene	C_15_H_24_	1.83 ± 0.26	1.95 ± 0.22
68	21.65	modephene	C_15_H_24_	1.22 ± 0.11	0.75 ± 0.18
69	21.85	berkheyaradulene	C_15_H_24_	1.66 ± 0.24	0.67 ± 0.11
70	22.00	β-farnesene	C_15_H_24_	0.87 ± 0.05	0.65 ± 0.09
71	22.11	acoradiene	C_15_H_24_	0.74 ± 0.03	0.62 ± 0.07
72	22.17	dihydropseudoionone	C_13_H_22_O	0.24 ± 0.01	0.47 ± 0.09
73	22.36	γ-elemene	C_15_H_24_	21.76 ± 1.78	19.08 ± 1.03
74	22.67	caryophyllene	C_15_H_24_	6.88 ± 0.64	5.28 ± 0.51
75	22.78	selina-5,11-diene	C_15_H_24_	3.43 ± 0.42	1.88 ± 0.84
76	22.92	elixene	C_15_H_24_	7.60 ± 0.60	7.18 ± 0.78
77	23.35	γ-curcumene	C_15_H_24_	1.60 ± 0.10	1.17 ± 0.15
78	23.49	α-curcumene	C_15_H_22_	3.05 ± 0.09	0.30 ± 0.03
79	23.55	α-farnesene	C_15_H_24_	2.42 ± 0.19	0.55 ± 0.76
80	23.74	1,5,9,9-tetramethyl-1,4,7-cycloundecatriene	C_15_H_24_	4.47 ± 0.27	3.28 ± 0.40
81	24.01	nootkatene	C_15_H_22_	1.03 ± 0.06	1.63 ± 0.37
82	24.23	β-bisabolene	C_15_H_24_	8.18 ± 0.39	6.47 ± 1.10
83	24.61	α-amorphene	C_15_H_24_	0.78 ± 0.06	0.75 ± 0.11
84	24.83	β-selinene	C_15_H_24_	25.43 ± 1.33	19.85 ± 2.55
85	24.97	α-selinene	C_15_H_24_	—	1.60 ± 0.27
86	25.10	(3E,5E)-7-isopropyl-8-methyl-3,5,7-nonatrien-2-one	C_13_H_20_O	6.62 ± 0.36	4.19 ± 0.96
87	25.27	cadina-1(10),4-diene	C_15_H_24_	1.60 ± 0.13	2.16 ± 0.24
88	25.47	cuparene	C_15_H_22_	1.99 ± 0.13	1.64 ± 0.17
89	25.66	6*S*,10*R*-dimethylbicyclo[4.4.0]decan-1-en-3-one	C_12_H_18_O	2.58 ± 0.12	1.93 ± 0.20
90	25.92	guaia-3,9-diene	C_15_H_24_	2.96 ± 0.12	2.22 ± 0.22
91	26.07	β-cadinene	C_15_H_24_	1.74 ± 0.05	1.70 ± 0.21
92	26.42	selina-3,7(11)-diene	C_15_H_24_	36.63 ± 0.97	36.61 ± 0.85
93	26.60	germacrene B	C_15_H_24_	13.62 ± 0.38	12.74 ± 0.97
94	27.01	β-vatirenene	C_15_H_22_	0.54 ± 0.02	0.38 ± 0.06
95	27.16	(4aR,8aS)-4a-methyl-1-methylene-7-(propan-2-ylidene)decahydronaphthalene	C_15_H_24_	0.88 ± 0.06	0.71 ± 0.06
96	27.50	γ-vetivenene	C_15_H_22_	8.21 ± 0.20	7.27 ± 0.29
97	27.84	3,5,11-eudesmatriene	C_15_H_22_	0.48 ± 0.02	0.37 ± 0.04
98	28.58	caryophyllene oxide	C_15_H_24_O	3.82 ± 0.19	2.20 ± 0.45
99	29.43	2-(1-methylethylidene)octahydro-4H-inden-4-one	C_12_H_18_O	2.07 ± 0.13	1.32 ± 0.20
100	29.58	humulene 6,7-epoxide	C_15_H_24_O	1.68 ± 0.06	1.03 ± 0.19
101	29.62	isospathulenol	C_15_H_24_O	—	0.49 ± 0.13
102	29.88	spathulenol	C_15_H_24_O	1.79 ± 0.09	1.22 ± 0.19
103	30.25	ledene oxide-(II)	C_15_H_24_O	1.32 ± 0.10	0.89 ± 0.15
104	30.42	caryophylla-4(12),8(13)-dien-5β-ol	C_15_H_24_O	0.99 ± 0.04	0.61 ± 0.14
105	31.01	β-selinenol	C_15_H_26_O	1.62 ± 0.11	1.31 ± 0.19
106	31.46	atractylon	C_15_H_20_O	100.41 ± 2.62	81.83 ± 6.35
107	31.96	aristolone	C_15_H_22_O	0.49 ± 0.05	0.62 ± 0.08
108	32.09	tetradecanoic acid	C_14_H_28_O_2_	0.72 ± 0.03	0.84 ± 0.39
109	32.18	juniper camphor	C_15_H_26_O	1.27 ± 0.08	1.04 ± 0.25
110	32.85	6-isopropenyl-4,8a-dimethyldecahydro-1-naphthalenol	C_15_H_26_O	0.36 ± 0.03	0.28 ± 0.03
111	33.03	isonootkatol	C_15_H_24_O	1.69 ± 0.31	1.35 ± 0.16
112	33.93	2-(4a,8-dimethyl-1,2,3,4,4a,5,6,7-octahydro-octahydro-2-naphthalenyl)-2-propen-1-ol	C_15_H_24_O	0.65 ± 0.05	0.49 ± 0.06
113	34.27	neocnidilide	C_12_H_18_O_2_	1.39 ± 0.10	1.39 ± 0.26
114	34.57	dehydrofukinone	C_15_H_22_O	9.42 ± 0.69	10.32 ± 0.97
115	35.69	7-methyl-4-(1-methylethylidene)bicyclo[5.3.1]undec-1-en-8-ol	C_15_H_24_O	0.63 ± 0.08	0.52 ± 0.06
116	35.84	(1R,7S)-germacra-4(15),5,10(14)-trien-1β-ol	C_15_H_24_O	1.84 ± 0.18	1.05 ± 0.17
117	37.35	ethyl pentadecanoate	C_17_H_34_O_2_	0.15 ± 0.02	—
118	37.72	isovalencenyl formate	C_16_H_24_O_2_	0.51 ± 0.72	0.16 ± 0.02
119	38.03	6-[1-(hydroxymethyl)vinyl]-4,8a-dimethyl-1,2,3,5,6,7,8,8a-octahydro-2-naphthalenol	C_15_H_24_O_2_	0.34 ± 0.05	0.25 ± 0.02
120	39.48	palmitoleic acid	C_16_H_30_O_2_	1.30 ± 0.17	1.78 ± 0.49
121	40.00	hexadecanoic acid	C_16_H_32_O_2_	4.95 ± 0.32	4.47 ± 0.52
122	40.18	β-cyclocostunolide	C_15_H_20_O_2_	3.51 ± 0.21	1.39 ± 0.59
123	40.95	(E)-valerenyl isovalerate	C_20_H_32_O_2_	3.91 ± 0.53	2.55 ± 0.44
124	41.29	ethyl hexadecanoate	C_18_H_36_O_2_	1.43 ± 0.15	0.33 ± 0.11

### 3.3. Differentiation of volatiles in crude and processed Baizhu Shaoyao San

In the present work, some obvious changes of volatile components between crude and processed BSS were observed from the GC–MS chromatogram. A total of 120 compounds were identified as common peaks in both crude and processed BSS samples. Processing might significantly change the volatiles in BSS; thus, one compound that was detected in crude BSS samples disappeared in processed BSS samples, and three compounds were newly generated in BSS after processing.

**Figure 3.**
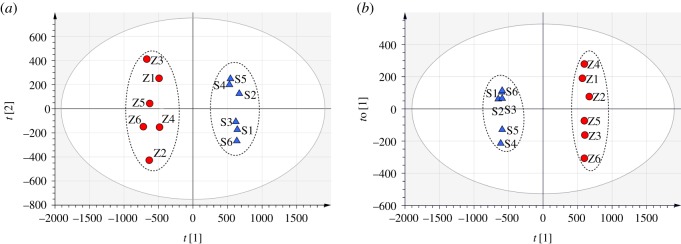
PCA score scatter plot (*a*) and OPLS-DA score scatter plot (*b*) based on global chemical profiling of volatiles from crude and processed BSS. (Triangles) crude BSS; (circles) processed BSS.

To comprehensively characterize the differences in volatile components between crude and processed BSS, multivariate data analysis was performed for the classification of these two groups. Unsupervised PCA was first employed to investigate if crude and processed BSS could be separated on the basis of their differences in volatile compounds. As shown in figure 3*a*, a total of six batches of crude and six batches of their corresponding processed BSS samples were distinguished from each other along PC1 in the two-dimensional PCA score scatter plot. Samples of crude BSS were characterized with positive scores, whereas samples of processed BSS showed negative scores, indicating that crude and processed BSS were distinctively different in their chemical patterns. The classification parameters of the PCA model revealed good fitness and predictability, as indicated by *R*^2^*X* (cum) and *Q*^2^ (cum) values of 0.777 and 0.608, respectively.

To further analyse the distinct components of the volatiles between crude and processed BSS, supervised OPLS-DA was subsequently applied to the GC–MS data matrices. As shown in figure 3*b*, the score scatter plot of the analysis demonstrated that all the test samples could be unambiguously grouped into two separated clusters along the discriminating *t* [1]: crude BSS gathered around the negative region, whereas processed BSS gathered around the positive region, suggesting a global difference in the composition of volatile compounds between two kinds of BSS. Moreover, all observation values decreased in the Hotelling T2 (0.95) ellipse, and the fitting parameters of this model were 0.716 of *R*^2^*X* (cum), 0.998 of *R*^2^*Y* (cum) and 0.983 of *Q*^2^ (cum), indicating that the OPLS-DA model was remarkably good in fitness and prediction.

**Figure 4.**
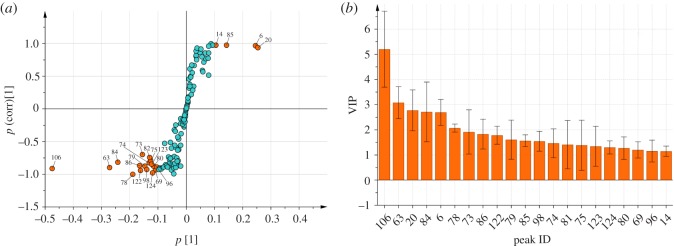
S-plot (*a*) and VIP (*b*) analyses associated with OPLS-DA score plot scatter. Numbers for peaks used are as given in table 2.

To identify the potential chemical markers that contribute to the difference between crude and processed BSS, the S-plot and VIP analyses were performed following the OPLS-DA. In the S-plot (figure 4*a*), each round point represented a compound with a peak number corresponding with table 1; the *X*-axis represented the contribution to the difference, wherein the farther the point is distributed from zero, the more the compound contributed to the difference between crude and processed BSS; the *Y*-axis represented the confidence to the difference, wherein the farther the point is distributed from zero, the higher the level of confidence that the compound contributed to the difference between crude and processed BSS. Therefore, the points scattering at both ends of ‘S' with *p* > 0.1 and *p*(corr) greater than 0.5 are marked red in figure 4*a*, which were thought to be the potential markers. Moreover, the VIP plot (figure 4*b*) also supported the result because all the marker compounds obtained from S-plot showed VIP values greater than 1. Finally, 21 volatile compounds with significant difference between crude and processed BSS were found, in which the contents of 4 components (peaks 6, 14, 20 and 85) remarkably increased in BSS after processing, and the contents of 17 components (peaks 63, 69, 73, 74, 75, 78, 79, 80, 82, 84, 86, 96, 98, 106, 122, 123, 124) remarkably decreased in processed BSS compared with crude samples. The results are summarized in table 2.

**Table 2.** Results of 21 marker compounds that can classify crude and processed BSS (*n* = 6).

no.	compound name	*p*	*p*(corr)	VIP
6	furfural	0.246	0.970	2.692
14	5-methyl-2-furfural	0.105	0.973	1.148
20	D-limonene	0.254	0.937	2.773
63	7,7-dimethyl-1-vinylbicyclo[2.2.1]heptan-2-one	−0.272	−0.896	3.079
69	berkheyaradulene	−0.110	−0.937	1.201
73	γ-elemene	−0.156	−0.700	1.915
74	caryophyllene	−0.131	−0.827	1.466
75	selina-5,11-diene	−0.126	−0.785	1.386
78	α-curcumene	−0.189	−0.998	2.069
79	α-farnesene	−0.146	−0.877	1.608
80	1,5,9,9-tetramethyl-1,4,7-cycloundecatriene	−0.116	−0.872	1.271
82	β-bisabolene	−0.129	−0.744	1.410
84	β-selinene	−0.242	−0.819	2.711
85	α-selinene	0.143	0.978	1.563
86	(3E,5E)-7-isopropyl-8-methyl-3,5,7-nonatrien-2-one	−0.164	−0.865	1.830
96	γ-vetivenene	−0.104	−0.893	1.154
98	caryophyllene oxide	−0.139	−0.924	1.546
106	atractylon	−0.475	−0.916	5.202
122	β-cyclocostunolide	−0.162	−0.938	1.782
123	(E)-valerenyl isovalerate	−0.123	−0.842	1.344
124	ethyl hexadecanoate	−0.119	−0.979	1.299

### 3.4. Analysis of proper processing mechanisms of some volatile components in Baizhu Shaoyao San

The contents of furfural and 5-methyl-2-furfural were significantly increased in processed BSS compared with crude BSS. According to the literature, these furfural series compounds can be generated from the Maillard reaction in the presence of amino compounds and reducing sugars in the field of food processing [31]. Similarly, in the processing of Chinese medicinal materials, the plausible mechanisms underlying these changes are that the amino acids and sugars found in medicinal and auxiliary materials may induce the Maillard reaction at high temperatures during stir-frying. Furthermore, these Maillard reaction products could produce a special aroma to stimulate appetite and modulate gut microbiota composition to promote digestion, which is consistent with the theory of TCM that processing BSS could invigorate and enhance the functions of spleen and stomach [32]. Atractylon, which is the main component of the dryness property that can cause impairments to the ‘yin' from the perspective of TCM [33], has the highest content in the volatiles of BSS and shows substantial decrease after processing. The mechanism, as summarized from literature, is that processing reduces the side effects of atractylon by oxidation reaction during stir-frying and transforms it into atractylenolide I, atractylenolide II and atractylenolide III (not detected in this experiment because of their non-volatile property), which are effective in tonifying the spleen, protecting the gastric mucosa and inhibiting inflammation [8,34,35]. Given the high contents of furfural, 5-methyl-2-furfural, atractylenolide I, atractylenolide II and atractylenolide III, and the low content of atractylon, the clinical prescription of processed BSS might be highly effective.

### 3.5. Comparison of needle trap device method with other conventional methods

Because the study of volatile oils of BSS using the NTD and other conventional methods has not been reported before, some relevant studies on the single herbs contained in BSS were employed to make brief comparisons between the NTD method and other conventional methods, which might not be comprehensive but provide some references for further investigation. Firstly, compared with steam distillation and headspace sampling method in the literature, our NTD method could trap a wider range of volatile compositions ranging from low to high boiling point. Specifically, steam distillation exhibited relatively higher concentrations of the high boiling point compounds and relatively lower concentrations of the low boiling point compounds in Atractylodis Macrocephalae Rhizoma, Paeoniae Radix Alba, Citri Reticulatae Pericarpium and Saposhnikoviae Radix, especially those heavy hydrocarbons (C*_n_* > 16) with more oxygens in their molecular formulas and those light hydrocarbons (C*_n_* < 8) [36–40]. A possible explanation for the phenomenon is the loss of volatiles with low boiling point and the occurrence of oxidation reaction during the high temperature extraction. On the contrary, headspace sampling method exhibited almost all peaks of the low and middle boiling point components and very few high boiling point components concluded from the published reports of Atractylodis Macrocephalae Rhizoma and Citri Reticulatae Pericarpium [36,41]. Secondly, the NTD method showed much higher enrichment efficiency and sensitivity compared with other conventional methods. For the NTD method in our study, only a small amount of the sample (0.085 g) was required to achieve the strong response in detection, while steam distillation needed a few hundred grams and headspace sampling needed approximately 1 g to meet the same goal. In addition, the NTD method could enable a good selectivity in sampling by applying a specific packing material or several packing materials in combination to obtain target components in complex matrix, which was almost impossible for other conventional methods.

## 4. Conclusion

In this study, GC–MS based on NTD technique combined with multivariate data analysis was established to characterize the chemical consistency and difference of volatiles in BSS before and after processing. Unlike the conventional approaches of phytochemical analysis, the proposed method could globally and rapidly profile the volatile components of crude and processed BSS and reveal the chemical markers between these two kinds of samples more efficiently and comprehensively. Our results suggested that the levels of volatiles were clearly distinguished between crude and processed BSS using PCA and OPLS-DA models; moreover, 21 compounds were identified as candidate markers that could be employed to quickly differentiate these two types of samples using S-PLOT and VIP analyses. These successful extraction, detection and identification of volatile markers for the discrimination of processed BSS from crude BSS suggested that the strategy might also be applicable for the investigation of chemical transformation underlying herb processing in other herbal formulae of TCM. Furthermore, the methods of composition analysis and statistical processing in this study could shed light on other similar studies on plants or medicinal herbs with geographical disparity, environmental variations and species differences.

## Supplementary Material

Figure S1. Profile of volatiles in blank sample obtained by GC-MS

## Data accessibility

The data supporting our paper have been deposited in Dryad Digital Repository (http://dx.doi.org/10.5061/dryad.6jg5g) [42].
