# Supplementary figures and images for: Discrimination of volatiles in herbal formula Baizhu Shaoyao San before and after processing using needle trap device with multivariate data analysis
Source: R Soc Open Sci. 2018 Jun 20;5(6):171987. doi: 10.1098/rsos.171987 (PMC6030309; doi:10.1098/rsos.171987)

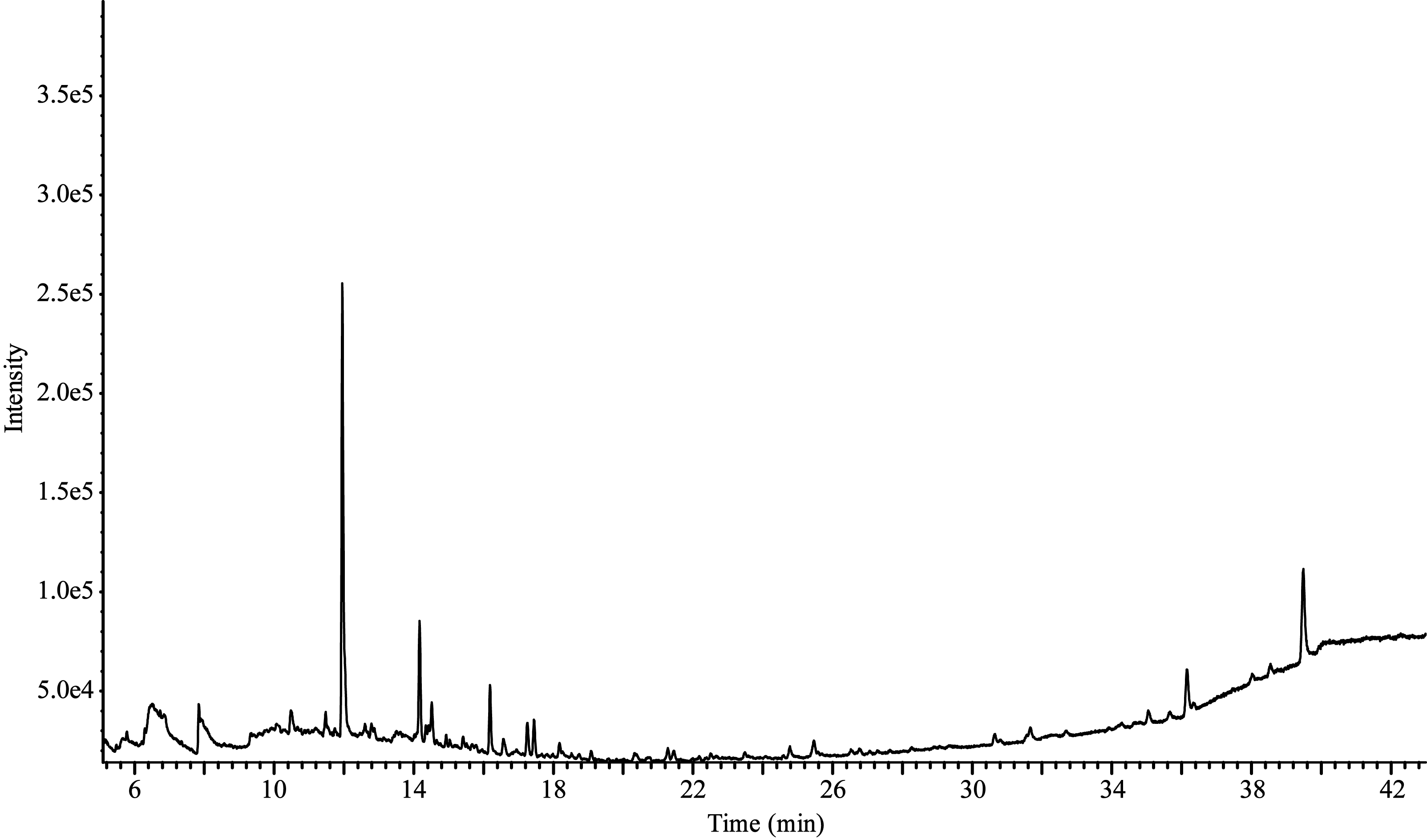

Supplement: Figure S1. Profile of volatiles in blank sample obtained by GC-MS [file rsos171987supp1.tif]
